# Identification of breast cancer cell subtypes sensitive to ATG4B inhibition

**DOI:** 10.18632/oncotarget.11408

**Published:** 2016-08-19

**Authors:** Svetlana Bortnik, Courtney Choutka, Hugo M. Horlings, Samuel Leung, Jennifer H. Baker, Chandra Lebovitz, Wieslawa H. Dragowska, Nancy E. Go, Marcel B. Bally, Andrew I. Minchinton, Karen A. Gelmon, Sharon M. Gorski

**Affiliations:** ^1^ The Genome Sciences Centre, BC Cancer Agency, Vancouver, BC, Canada; ^2^ Interdisciplinary Oncology Program, University of British Columbia, Vancouver, BC, Canada; ^3^ Department of Molecular Biology and Biochemistry, Simon Fraser University, Burnaby, BC, Canada; ^4^ Department of Pathology and Laboratory Medicine, University of British Columbia, Vancouver, BC, Canada; ^5^ Vancouver General Hospital, BC Cancer Agency, Vancouver, BC, Canada; ^6^ Department of Pathology, Netherlands Cancer Institute, Amsterdam, The Netherlands; ^7^ Radiation Biology Unit - Department of Integrative Oncology, BC Cancer Agency, Vancouver, BC, Canada; ^8^ Department of Experimental Therapeutics, BC Cancer Agency, Vancouver, BC, Canada; ^9^ Faculty of Pharmaceutical Sciences, University of British Columbia, Vancouver, BC, Canada; ^10^ Department of Pathology and Laboratory Medicine, University of British Columbia, Vancouver, BC, Canada; ^11^ Centre for Drug Research and Development, Vancouver, BC, Canada; ^12^ Medical Oncology, BC Cancer Agency, Vancouver, BC, Canada; ^13^ Department of Medicine, University of British Columbia, Vancouver, BC, Canada; ^14^ Centre for Cell Biology, Development, and Disease, Simon Fraser University, Burnaby, BC, Canada

**Keywords:** breast cancer, autophagy, ATG4B, HER2, trastuzumab

## Abstract

Autophagy, a lysosome-mediated degradation and recycling process, functions in advanced malignancies to promote cancer cell survival and contribute to cancer progression and drug resistance. While various autophagy inhibition strategies are under investigation for cancer treatment, corresponding patient selection criteria for these autophagy inhibitors need to be developed. Due to its central roles in the autophagy process, the cysteine protease ATG4B is one of the autophagy proteins being pursued as a potential therapeutic target. In this study, we investigated the expression of ATG4B in breast cancer, a heterogeneous disease comprised of several molecular subtypes. We examined a panel of breast cancer cell lines, xenograft tumors, and breast cancer patient specimens for the protein expression of ATG4B, and found a positive association between HER2 and ATG4B protein expression. We showed that HER2-positive cells, but not HER2-negative breast cancer cells, require ATG4B to survive under stress. In HER2-positive cells, cytoprotective autophagy was dependent on ATG4B under both starvation and HER2 inhibition conditions. Combined knockdown of ATG4B and HER2 by siRNA resulted in a significant decrease in cell viability, and the combination of ATG4B knockdown with trastuzumab resulted in a greater reduction in cell viability compared to trastuzumab treatment alone, in both trastuzumab-sensitive and -resistant HER2 overexpressing breast cancer cells. Together these results demonstrate a novel association of ATG4B positive expression with HER2 positive breast cancers and indicate that this subtype is suitable for emerging ATG4B inhibition strategies.

## INTRODUCTION

During the past decade, a tremendous effort has been devoted to better understanding the dynamic role of autophagy in different cancers, including breast cancer - the most common cancer in women worldwide (reviewed in [1–5]). However, little progress has been made in applying this knowledge clinically for diagnostic or therapeutic purposes. One of the outstanding challenges in the autophagy field is determining whether certain cancer types or subtypes are sensitive to specific autophagy-related inhibition strategies, with the longer term aim of better patient selection for relevant treatments.

Macroautophagy (herein referred to as autophagy), a lysosome-mediated degradation and recycling process, plays an important role in maintaining cell homeostasis and functions as an adaptive survival response to various cellular stresses, including hypoxia and chemotherapy [1]. Depending on the stage of tumorigenesis, autophagy may act as either a tumor suppressor [1, 6–9] or promoter [1, 10–12]. Numerous clinical trials targeting autophagy with the non-specific pharmacological inhibitors chloroquine (CQ) and hydroxychloroquine (HCQ) in various cancers are underway, and the first published results highlight the need for the development of reliable criteria for better patient selection [13, 14]. In addition, whereas autophagy inhibitors that target specific proteins crucial for distinct steps in the autophagic process are currently under development [15–21], our knowledge about the sensitivity of different cancers to these inhibition strategies is limited.

The cysteine protease ATG4B is a potential target for autophagy inhibition due to its important roles in the autophagic pathway. ATG4B is crucial for the processing of the microtubule-associated protein 1 light chain 3B (MAP1LC3B, or LC3B), a ubiquitin-like protein, which is required for autophagosome formation [22]. ATG4B is responsible for the cleavage of the carboxyl terminus of newly synthesized pro-LC3B to provide LC3-I, a reaction essential for further LC3B conjugation to the lipid phosphatidylethanolamine (PE) during autophagosome formation [23–25]. LC3B-PE (or LC3B-II) is widely used as a key autophagy marker [26]. ATG4B also delipidates LC3B to release the protein from membranes and recycles de-conjugated LC3B to maintain a reservoir of its unlipidated form for new autophagosome formation [22, 27]. Other ATG4 paralogs (ATG4A, ATG4C, ATG4D) have also been described in mammalian cells [28, 29]. Each of the four ATG4 family members displays differing substrate affinities for the various mammalian ATG8 family members (including MAP1LC3B), allowing potential fine-tuning of the autophagic process [30, 31]. Notably, ATG4B displays the broadest substrate specificity compared to the other ATG4 family members [29–31].

ATG4B appears to act as a positive regulator of proliferation in some cancer types [32, 33], although the roles of ATG4B and the effects of ATG4B inhibition are cell type, treatment, and context-dependent [16, 32–34]. Previous reports showed elevated ATG4B expression in colorectal tumor cells [32] and lung cancer cells [35] compared with adjacent normal cells. However, little is known about ATG4B expression and function in breast cancers, nor have the contexts been identified where ATG4B inhibition might be beneficial.

Approximately 15%–20% of breast cancers have amplification of the *ERBB2* gene, which codes for HER2 (human epidermal growth factor receptor 2) on chromosome 17 [36]. Patients with this subtype of breast cancer historically had more aggressive disease and worse outcomes compared to patients with some other breast cancer subtypes. Since approval in 1998 of the first anti-HER2 agent (trastuzumab) and development of molecularly targeted therapies for HER2-positive breast cancer, disease outcomes have significantly improved [36], although drug resistance remains a challenge [37, 38]. Previous studies [39, 40] showed that autophagy inhibition with pharmacological inhibitors CQ or HCQ may help overcome resistance to anti-HER2 therapy. However, the role of ATG4B and the effects of ATG4B inhibition in HER2-positive breast cancers have never been reported before.

Here we evaluated ATG4B protein expression in a panel of HER2 negative and HER2 positive breast cancer cell lines. Unexpectedly, we found that ATG4B expression was elevated in HER2-positive breast cancer cells. We further evaluated the function of ATG4B in these cells and found that HER2-positive breast cancer cells, but not HER2-negative breast cancer cells required ATG4B to survive under stress. Importantly, we showed that ATG4B inhibition sensitized HER2-positive breast cancer cells to anti-HER2 treatment.

## RESULTS

### ATG4B protein expression correlates with HER2 status in breast cancer cell lines

**Figure 1:**
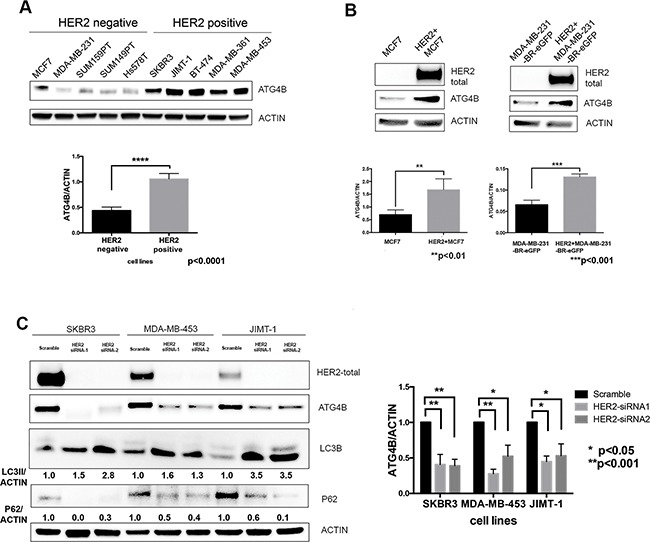
ATG4B protein expression correlates with HER2 status **A.** HER2-positive cell lines have higher protein levels of ATG4B as compared to HER2-negative cell lines. Representative western blot analysis shows ATG4B basal expression in a panel of HER2-positive (n=5) and HER2-negative (n=5) breast cancer cell lines. Bar plots demonstrate average ATG4B expression within each group of cell lines (mean±SEM) normalized to actin (used as internal control for protein loading); n=3; *P* values are based on the Student's *t*-test. **B.** HER2+ overexpression in MCF7 and MDA-MB-231-BR-eGFP breast cancer cells results in elevated ATG4B protein levels. Representative western blot and bar plots show ATG4B expression normalized to actin loading control (mean±SEM; n=3); *P* values are based on the Student's *t*-test. **C.** HER2 knockdown in three HER2-overexpressing cell lines (SKBR3, MDA-MB-453, and JIMT1) results in a decrease in ATG4B protein levels, increase in LC3B-II and decrease in SQSTM1/p62 expression. Representative western blot from 3 independent experiments. Bar graph on right shows ATG4B expression normalized to actin loading control; (mean±SEM; n=3); *P* values are based on the one-way ANOVA with Dunnett post-test.

We compared basal levels of ATG4B protein expression in five HER2 positive and five HER2 negative breast cancer cell lines, and found that ATG4B levels were significantly (p<0.0001) elevated in HER2 positive cells (Figure 1A). To further determine whether the observed cell line differences in ATG4B levels can be attributed to HER2 status alone, we employed genetic approaches to specifically modify HER2 status in cells with different genetic backgrounds. Overexpression of HER2 in HER2-negative MCF7 and MDA-MB-231-BR-eGFP cells (Figure 1B) resulted in a significant increase in ATG4B protein expression (p<0.01). Conversely, HER2 knockdown using siRNA treatment in three HER2-positive cell lines (SKBR3, MDA-MB-453, and JIMT- 1) led to a significant decrease in ATG4B levels (Figure 1C). Together, these findings support a positive association between HER2 and ATG4B protein levels in breast cancer.

To determine if the expression of other autophagy proteins correlated with HER2 status, we examined ATG5, ATG7, BECN1/Beclin 1 and the other ATG4 family members in the cell line panel. We observed no significant correlations between protein expression level and HER2 status (Supplementary Figure S1); there was a trend towards higher protein expression of Beclin 1 in HER2 positive cells, but the difference was not statistically significant. To determine if ATG4B mRNA levels correlated with HER2 status, we queried mRNA data from The Cancer Genome Atlas consortium. RNA-seq derived mRNA levels for the ATG4 paralogs in patients with invasive breast carcinoma (BRCA) were not found to be dynamic between patient groups that differ in ERBB2/HER2 status, including stratification by PAM50 subtype (n=579), by DNA alteration status (amplifications and/or mutations versus wild type; n=959) or by *ERBB2* amplification, mRNA overexpression (OE), and/or protein OE versus median expression (n=410) (Supplementary Figure S1). Similarly, there was no correlation between ATG4B mRNA levels and ERBB2/HER2 protein levels across all TCGA BRCA patients assessed for ERBB2/HER2 protein abundance (n=410; Spearman r=−0.023); however, the correlation does increase in patients with either ERBB2/HER2 amplification, mRNA OE, and/or protein OE (n=72; Spearman r=0.30) (Supplementary Figure S1). Together, these results suggest that the regulation of ATG4B levels occurs post-transcriptionally.

### HER2 status is inversely associated with autophagic activity

**Figure 2:**
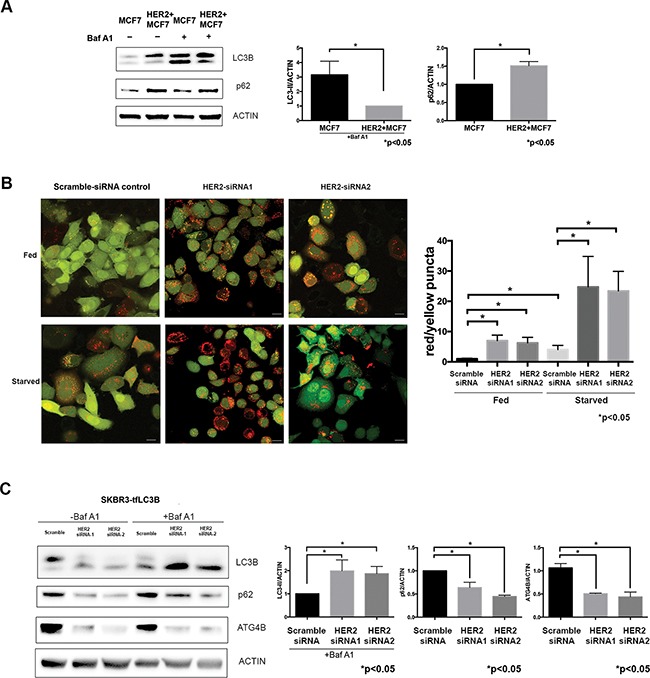
HER2 status inversely correlates with autophagic activity **A.** HER2-overexpressing cells have decreased basal autophagy levels. Western blot flux assay using saturating (60nM) concentrations of Bafilomycin A1 (Baf A1) shows LC3B-II and p62 levels in parental MCF7 cells as compared to HER2-overexpressing MCF7 cells. Bar graphs on right show LC3B-II (in the presence of Baf A1) and p62 expression, normalized to actin loading control (fold change); mean±SEM; n=3; *P* values are based on Student's t-test. **B.** HER2 knockdown increases autophagic flux. SKBR3 cells stably expressing mRFP-EGFP-LC3B protein were treated with either HER2 or scramble siRNA under fed and starved conditions. Increase in red puncta (autolysosomes) relative to yellow puncta (autophagosomes) indicates increased autophagic flux in response to HER2 knockdown. Bar graphs show average (mean±SEM) ratio of red to yellow puncta per cell. Data was collected from 3 independent experiments, and *P* values are based on the one-way ANOVA with Dunnett post-test. Scale bar, 50 μm. **C.** HER2 knockdown increases autophagic flux. Western blot flux assay using saturating (60nM) concentrations of Bafilomycin A1 (Baf A1) shows LC3B-II, p62, and ATG4B levels in cells treated with HER2 siRNAs as compared to scramble siRNA control. Bar graphs on right show LC3B-II (in the presence of Baf A1), p62, and ATG4B expression, normalized to actin loading control (fold change); mean±SEM; n=3; *P* values are based on Student's t-test.

To determine how HER2-positivity affects autophagic activity, we compared autophagic flux under basal conditions in MCF7 and HER2+MCF7 cells using the late-stage autophagy inhibitor Bafilomycin A1 [26] and the results of these studies are summarized in Figure 2A. There was an accumulation of LC3B-I and reduction of LC3B-II in HER2+MCF7 cells in the presence of Bafilomycin A1 as compared to parental MCF7 cells (Figure 2A), consistent with increased LC3B-II recycling by ATG4B and decreased autophagic flux.

HER2 knockdown resulted in an increase in LC3-II levels together with a decrease in p62 levels in all tested cell lines (Figure 1C), which suggested an increase in autophagic flux. To monitor autophagic flux in response to HER2 knockdown, we used mRFP-eGFP tandem fluorescent-tagged LC3B (tfLC3B) and fluorescence microscopy – an assay based on differential pH stability of eGFP and mRFP fluorescent proteins [26, 41], which allows the simultaneous evaluation of autophagy induction and flux through autophagic compartments without requiring the use of lysosomal inhibitors [26]. SKBR3-tfLC3B (HER2 positive) cells were treated with HER2 siRNA, and using green/red merged images, we quantitated yellow (i.e., RFP+GFP+, autophagosomes), and red (i.e., RFP+GFP-, autolysosomes) puncta per cell. As shown in Figure 2B, there was a significant increase in both red and yellow puncta following HER2 knockdown even under basal (i.e. fed) conditions, which confirms an induction of autophagy and increase in autophagic flux. Starvation, a commonly used stressor to induce autophagic flux [42], was used as a positive control. As seen in Figure 2B, an increase in autophagic activity in fed HER2 knockdown cells was comparable to that in starved control cells. Starvation in HER2-knockdown cells resulted in further increase of autophagic flux as seen by significantly higher red/yellow puncta ratio. An alternative autophagic flux assay, using a saturating concentration of lysosomal inhibitor Bafilomycin A1 in the same cell line, further confirmed the increase in autophagic flux (Figure 2C) in response to HER2 knockdown.

### HER2 positive cells require ATG4B for autophagy upregulation and survival under stress

**Figure 3:**
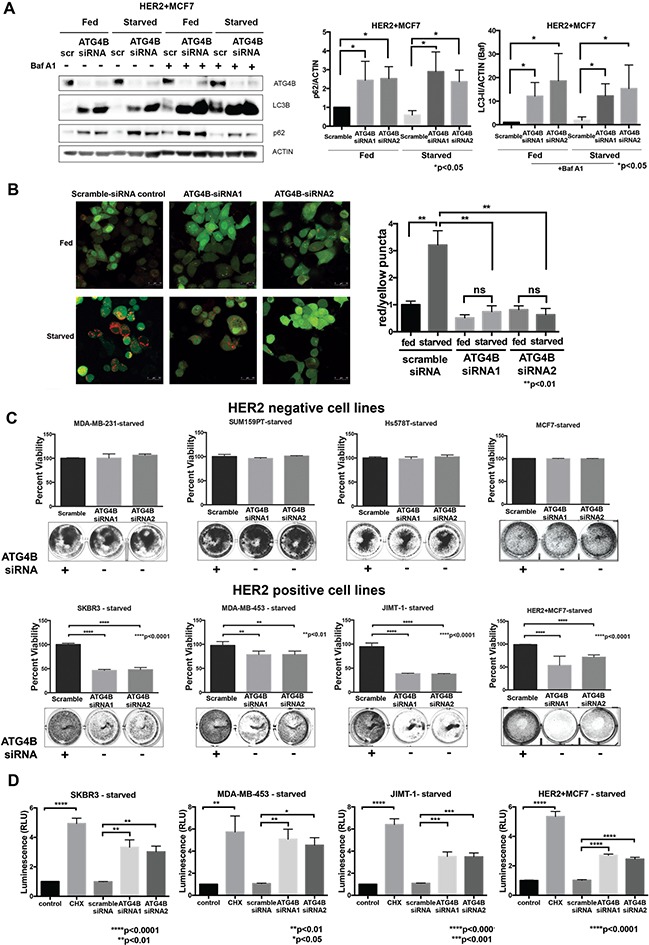
ATG4B is required for increased autophagic flux and survival under starvation in HER2 positive cells **A.** ATG4B knockdown results in inhibition of autophagy. Western blot flux assay using saturating concentrations of Bafilomycin A1 (Baf A1) show LC3B-II and p62 levels in HER2+MCF7 cells following ATG4B knockdown under fed and starved conditions. Bar graphs on right show LC3B-II (in the presence of Baf A1) and p62 expression, normalized to actin loading control (fold change); mean±SEM; n=3; *P* values are based on Student's t-test. **B.** ATG4B knockdown prevents HER2 positive cells from increasing autophagic flux in response to starvation. SKBR3 cells stably expressing mRFP-EGFP-LC3B protein were treated with either ATG4B or scramble siRNA under fed and starved conditions. Bar graphs show average (mean±SEM) ratio of red to yellow puncta per cell. Data was calculated from 3 independent experiments, and *P* values are based on the one-way ANOVA with Dunnett post-test. Scale bar, 50 μm. **C.** HER2-overexpressing cells are sensitive to ATG4B inhibition under starvation. HER2-negative (MDA-MB-231, SUM159PT, Hs578T, and MCF7) and HER2-positive (SKBR3, MDA-MB-453, JIMT-1, and HER2+MCF7) cells were treated twice with ATG4B siRNA under starved conditions, and colonies were visualized with crystal violet staining. Retained crystal violet staining was measured by absorbance at 590nm (A590) to generate a proliferation index. Bar plot indicates mean±SEM values from 3 independent experiments normalized to fed scramble controls. *P* values are based on the one-way ANOVA with Dunnett post-test. **D.** ATG4B knockdown under starvation results in caspase-dependent cell death in HER2-overexpressing cells. SKBR3, MDA-MB-453, JIMT-1, and HER2+MCF7 cells were assayed for induction of caspase-3/7 activity using the luminescence (RLU, relative luminescence unit; y axis)-based Caspase-Glo assay. Bar plots show mean±SEM values from 3 independent experiments. Cycloheximide (CHX) was used as a positive control for caspase activation. *P* values are based on the one-way ANOVA with Dunnett post-test.

To determine whether ATG4B knockdown results in changes in autophagic flux in HER2 positive cells, we performed a western blot-based autophagic flux assay, using saturating concentrations of Bafilomycin A1 in HER2+MCF7 cells (Figure 3A). Cells treated with ATG4B siRNA showed higher accumulation of LC3B-II as compared to scramble controls, consistent with a reduction in the recycling (de-conjugating) function of ATG4B [22, 27]. The observed accumulation of p62 (Figure 3A) supports an impairment of autophagy following ATG4B knockdown. These changes in LC3B-II and p62 were observed in both basal (i.e. fed) and starved conditions (Figure 3A). Similar results were observed in the HER2 positive cell line SKBR3 (Supplementary Figure S2).

To confirm suppression of autophagy following ATG4B knockdown, we used the alternative mRFP-eGFP-LC3B flux assay. In control scramble-siRNA treated SKBR3-tfLC3B cells, we found that starvation caused a substantial increase in autophagic flux as indicated by an increase in red puncta. However, there was no increase in red puncta in ATG4B-siRNA treated cells (Figure 3B). Moreover, there was an increase in yellow puncta in ATG4B-siRNA treated cells, consistent with defective de-conjugation of LC3B from the membranes. Together, these results show that ATG4B is required for starvation-induced autophagic flux.

To evaluate the effects of ATG4B inhibition on cell viability, HER2 positive and HER2 negative cells were treated with ATG4B siRNA under fed and starved (24 hours) conditions. While ATG4B knockdown had no significant effect on cell viability in fed conditions, it resulted in a significant (p<0.0001) decrease in the surviving cell fraction of HER2 positive cells under starvation – an effect not seen in HER2 negative cells (Figure 3C).

To determine whether ATG4B inhibition under starvation triggers caspase-dependent cell death in HER2 positive breast cancer cells, we assessed executioner caspase activity levels using the luminescence-based Caspase-Glo assay. Cycloheximide (CHX) was used as a positive control for induction of caspase activity. The results in Figure 3D show that ATG4B knockdown under starvation leads to increased levels of caspase activity compared with the scramble-siRNA control in all tested HER2 positive cell lines. These findings indicate that these HER2-positive cells require ATG4B for survival under starvation.

### ATG4B is required for upregulation of cytoprotective autophagy in response to HER2 knockdown

**Figure 4:**
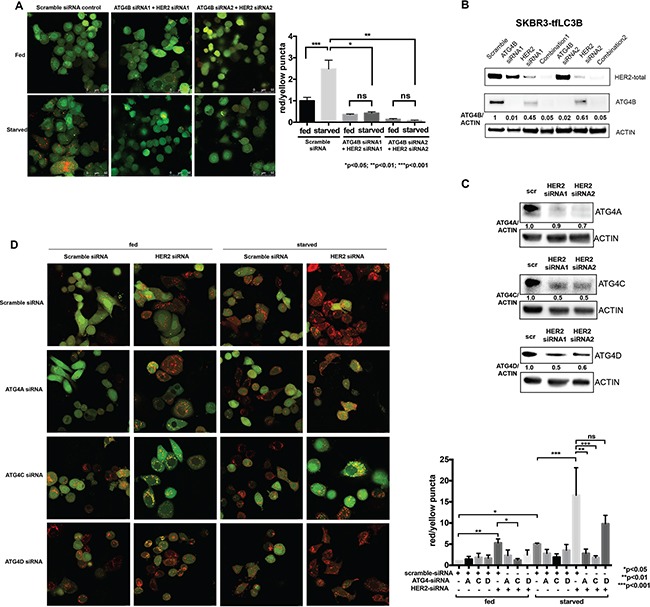
ATG4B, as well as ATG4C and ATG4A, contribute to the increased autophagic flux following HER2 knockdown **A.** Combination ATG4B and HER2 knockdown results in autophagy flux inhibition in HER2-positive cells. SKBR3 cells stably expressing mRFP-EGFP-LC3B protein were treated with HER2 and ATG4B siRNA (ATG4B siRNA1, center; ATG4B siRNA2, right) or scramble siRNA (control, left) under fed and starved conditions. Bar graphs show average (mean±SEM) ratio of red to yellow puncta per cell. Data shown is from 3 independent experiments, and *P* values are based on the one-way ANOVA with Dunnett post-test. Scale bar, 50 μm. **B.** HER2 knockdown results in decreased ATG4B levels, whereas ATG4B and ATG4B+HER2 knockdowns lead to a further decrease in ATG4B levels. Representative western blot analysis shows ATG4B levels in SKBR3-tfLC3B cells following ATG4B and HER2 knockdowns alone or combined. **C.** HER2 knockdown results in decreased ATG4A, ATG4C and ATG4D levels. Representative (n=2) western blot analysis shows ATG4A, ATG4C, and ATG4D levels in SKBR3-tfLC3B cells following HER2 knockdown. **D.** ATG4C (under both fed and starved conditions) and ATG4A (under starvation only) are required for autophagy induction following HER2 knockdown. SKBR3 cells stably expressing mRFP-EGFP-LC3B protein were treated with ATG4A, ATG4C, ATG4D or scramble siRNA alone or in combination with HER2 siRNA under fed and starved conditions. Bar graphs show average (mean±SEM) ratio of red to yellow puncta per cell. Data shown is from 3 independent experiments, and *P* values are based on the one-way ANOVA with Dunnett post-test. Scale bar, 10 μm.

We observed increased autophagic flux in response to HER2 inhibition alone, as well as dependency of HER2 positive cells on ATG4B for autophagy upregulation and survival under stress (starvation). These findings led us to pose the question of whether ATG4B is required for the increased autophagy following HER2 inhibition. In order to address this question, we performed a combination knockdown of HER2 and ATG4B in SKBR3-tfLC3B cells. As shown in Figure 4A, addition of ATG4B knockdown to HER2 knockdown prevented autophagy induction that was otherwise seen in cells treated with HER2-siRNA alone (Figure 2B). This effect was observed in both fed and starved conditions. Furthermore, combination knockdown of ATG4B and HER2 resulted in the inability of cells to upregulate autophagy in response to starvation, as seen by a significant (p<0.05) decrease in red puncta in combination (ATG4B and HER2) siRNA-treated cells compared to starved controls. This result indicates that ATG4B is required for the increase in autophagic flux in response to HER2 knockdown alone and for starvation-induced autophagy.

To address the question of why HER2 knockdown cells were able to induce autophagy despite having reduced ATG4B levels (Figures 1C, 2B, and 2C), whereas the combination (HER2 and ATG4B) knockdown cells were unable to induce autophagy (Figure 4A), we compared ATG4B levels by western blot analysis (Figure 4B). While HER2-knockdown alone resulted in a substantial reduction of ATG4B, the ATG4B levels were still relatively high compared to the ATG4B-knockdown alone or combination-knockdown cells (Figure 4D). The remaining ATG4B in HER2-knockdown alone cells was likely sufficient to enable autophagic flux, while the additional knockdown with ATG4B siRNA brought ATG4B to critically low levels, insufficient to promote cytoprotective autophagy.

Another explanation for increased autophagic flux following HER2 knockdown despite decreased ATG4B levels may come from the possible compensatory roles of other ATG4 family members. Western blot analysis (Figure 4C) revealed significant (similar to ATG4B) reduction in ATG4A, ATG4C, and ATG4D levels following HER2 knockdown. To test whether the remaining levels of ATG4A, ATG4C, and/or ATG4D function to upregulate autophagy following HER2 knockdown, we knocked down each of the ATG4 family members alone or in combination with HER2 knockdown under fed and starved conditions. Autophagic flux was assessed using the mRFP-eGFP-LC3B flux assay in SKBR3-tfLC3B cells. As shown in Figure 4D, ATG4C was involved in autophagy upregulation following HER2 knockdown under both fed and starved conditions, whereas ATG4A was only required under starvation. There was no apparent role for ATG4D in either condition. Together, these findings suggest that ATG4C and ATG4A may act to compensate for the decreased ATG4B levels and to help carry out the increased autophagic flux following HER2 knockdown.

### ATG4B knockdown sensitizes HER2-positive breast cancer cells to trastuzumab

**Figure 5:**
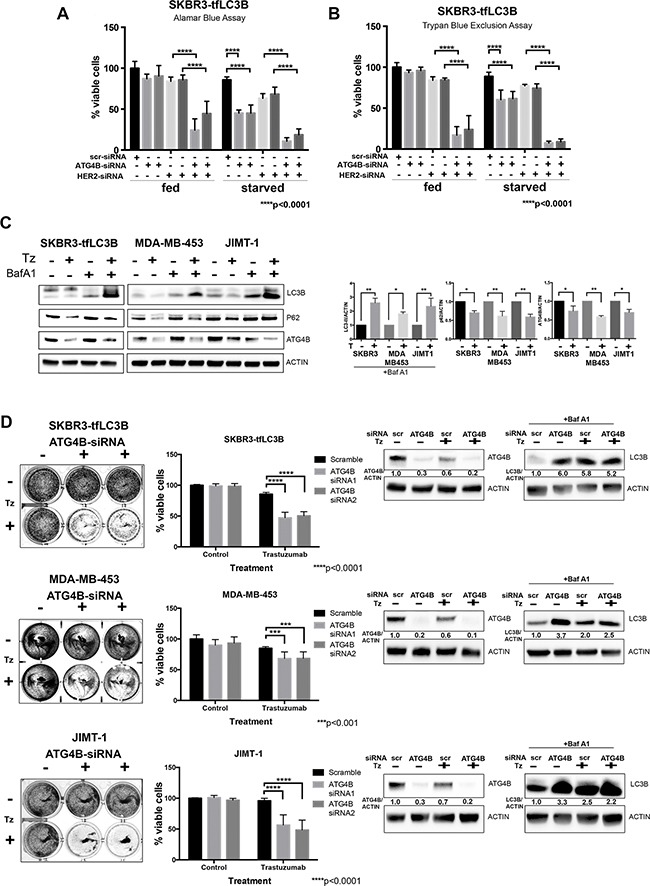
ATG4B knockdown sensitizes HER2 positive cells to HER2 inhibition **A.** and **B.** ATG4B is required for cell survival under HER2 inhibition. Combination ATG4B and HER2 knockdown results in decreased cell viability as compared to single knockdowns under fed and starved conditions. Alamar blue (A) and Trypan Blue exclusion (B) assays were used to assess cell viability following siRNA treatment. Bar graphs represent mean±SEM values of %viable cells relative to fed scramble controls (n=3 independent experiments). *P* values are based on the one-way ANOVA with Dunnett post-test. **C.** Trastuzumab (Tz) treatment results in increased autophagic flux and decreased levels of ATG4B. Representative western blots for SKBR3-tfLC3, MDA-MB-453, and JIMT-1 cells. Bar graphs on right show LC3B-II (in the presence of Baf A1), p62, and ATG4B expression, normalized to actin loading control (fold change); mean±SEM; n=3; *P* values are based on Student's t-test. **D.** ATG4B knockdown sensitizes HER2-positive cells to trastuzumab (Tz). SKBR3, MDA-MB-453, and JIMT-1 cells were pre-treated with ATG4B and scramble siRNAs, and then treated with trastuzumab for 72 hours. Colonies were visualized with crystal violet staining (on the left). Retained crystal violet staining was measured by A590 to generate a proliferation index (central panel). Bar plot indicates mean±SEM values from 3 independent experiments normalized to scramble siRNA controls. *P* values are based on the one-way ANOVA with Dunnett post-test. Accompanying representative western blot analysis of ATG4B levels, as well as flux assay of LC3B-II levels using saturating concentrations of Bafilomycin A1 (Baf A1) is shown on the right.

Evaluation of cell viability following combination knockdown of HER2 and ATG4B using the Alamar Blue assay (Figure 5A) showed a significant (p<0.0001) decrease in cell viability, which suggests that ATG4B is required for cell survival following HER2 knockdown. This finding was confirmed using the Trypan blue exclusion assay (Figure 5B), an alternative cell viability assay.

To investigate whether ATG4B inhibition can sensitize HER2-positive cells to available anti-HER2 therapy, we first tested whether treatment with trastuzumab (Herceptin) had similar effects on autophagy and ATG4B levels as did HER2 knockdown. We treated trastuzumab-sensitive SKBR3-tfLC3 cells with 10 ug/ml of trastuzumab, as well as trastuzumab-resistant MDA-MB-453 and JIMT-1 cells with a higher concentration of 100 ug/ml of trastuzumab, for 72 hours under fed conditions. An increase in autophagic flux in response to trastuzumab treatment was observed in all tested cell lines, as indicated by higher accumulation of LC3B-II in cells treated with the combination of trastuzumab and Bafilomycin A1, as compared to Bafilomycin A1 alone (Figure 5C). In addition, there was a reduction in p62 levels in trastuzumab-treated cells, consistent with increased autophagic flux. Moreover, similar to HER2 knockdown, trastuzumab caused a decrease in ATG4B levels in all tested cell lines.

We assessed cell viability by pre-treating SKBR3, MDA-MB-453, and JIMT-1 cells with ATG4B-siRNA and 48 hours later performed a second ATG4B knockdown, followed by incubation in trastuzumab for 72 hours. All cell lines, independent of their initial sensitivity to trastuzumab, showed a significant (p<0.001) decrease in cell viability following combination treatment with ATG4B-siRNA (Figure 5D) as compared to trastuzumab treatment with the scramble-siRNA. Results from western blot analysis of ATG4B and LC3B levels for this experiment (Figure 5D) were consistent with our previous observations (i.e. reduction in ATG4B levels and accumulation of LC3B following HER2 inhibition with trastuzumab). Taken together, these findings indicate that ATG4B inhibition can be an effective strategy to sensitize HER2-positive breast cancer cells to anti-HER2 therapy.

### ATG4B protein expression correlates with HER2 status in vivo

**Figure 6:**
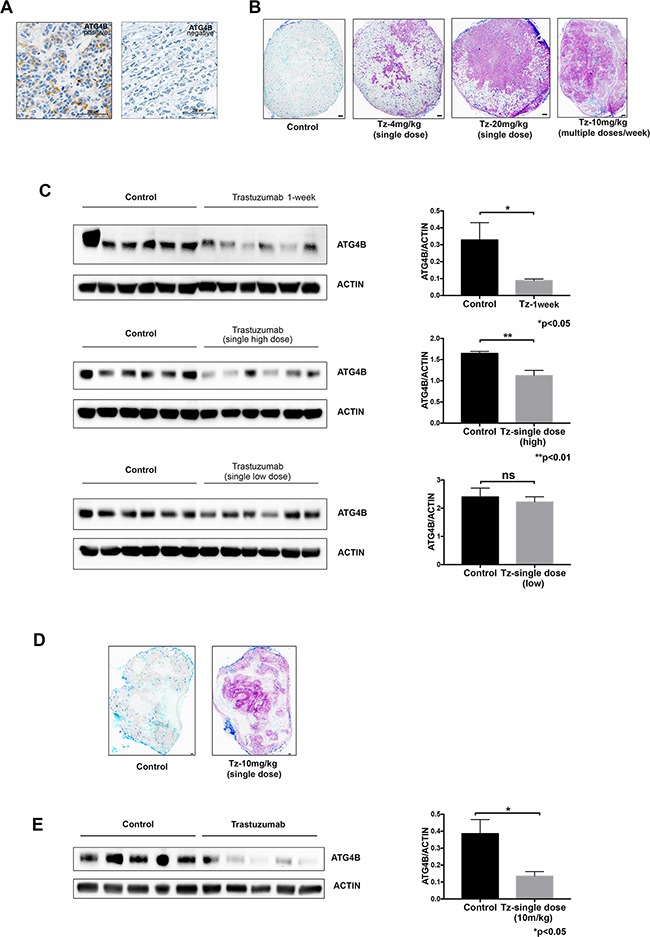
ATG4B levels correlate with HER2 status and trastuzumab treatment *in vivo* **A.** Immunohistochemical images representing patient breast tumors positive and negative for ATG4B expression. **B.** Distribution of trastuzumab through breast cancer xenografts in a dose-dependent manner. Mice bearing BT-474 xenograft tumors received single 4, 10 or 20 mg/kg doses of trastuzumab for 8h or 10 mg/kg every 72h for one week, with tissues collected 24 h after last dose. Bound trastuzumab was labeled in tumor cryosections using fluorescently tagged antihuman secondary antibodies; representative sections are shown with trastuzumab (magenta), CD31 (dark blue), and perfusion dye, DioC7(3) (cyan). Scale bar, 150 μm. **C.** Trastuzumab treatment results in reduction of ATG4B protein expression in BT-474 tumor xenografts in a dose-dependent manner. ATG4B and actin protein levels in tumors from the previously described experiment (panel B) were assessed with western blot analysis. Bar plots demonstrate average ATG4B expression within each treatment group (mean±SEM) normalized to actin (used as internal control for protein loading); *P* values are based on the Student's t-test. **D.** Distribution of trastuzumab in JIMT1 xenograft tumor collected 24 hour following treatment with a single dose of 10mg/kg trastuzumab. Bound trastuzumab was labeled in tumor cryosections using fluorescently tagged antihuman secondary antibodies; a representative section is shown with trastuzumab (magenta), CD31 (dark blue), and perfusion dye, DioC7(3) (cyan). Scale bar, 150 μm. **E.** Trastuzumab treatment results in reduction of ATG4B protein expression in JIMT1 tumor xenografts. ATG4B and actin protein levels in tumors were assessed with western blot analysis. Bar plots demonstrate average ATG4B expression within each treatment group (mean±SEM) normalized to actin (used as internal control for protein loading); *P* values are based on the Student's t-test.

To investigate the potential clinical relevance of our findings, we performed an exploratory analysis of breast cancer patient specimens (Table 1, Figure 6A) and found that ATG4B positivity was observed in a higher fraction of HER2 positive specimens as compared to HER2 negative samples (21% vs. 6% ATG4B positive, respectively; p=0.0204; Table 2). ATG4B positive expression was also significantly associated with grade 3 tumors, 10% of which were ATG4B positive, in contrast to grade 1 and 2 tumors, of which only 4% were ATG4B positive (p=0.0359, Table 2).

**Table 1:** Cohort characteristics

Cohort characteristics stratified by ATG4B	Total	ATG4B cytoplasmic score		
		negative	positive	missing
**Total**	330 (100%)	269 (82%)	22 (7%)	39(12%)
**Follow-up (years)**				
mean	12 ± 0	12 ± 0	14 ± 1	13±1
median	13	13	14	14
interquartile range	11 to 15	11 to 14	11 to 16	11.4 - 15.8
range (min to max)	1.1 to 25	1.1 to 23	6.7 to 23	1 to 25
missing	0	0	0	0
**%Age (group)**				
<40	28	22(79%)	1(4%)	5(18%)
40-49	98	82(84%)	7(7%)	9(9%)
50-65	130	108(83%)	9(7%)	13(10%)
>65	74	57(77%)	5(7%)	12(16%)
missing	0	0(0%)	0(0%)	0(0%)
**Grade**				
Grade 1	62	51(82%)	2(3%)	9(15%)
Grade 2	129	107(83%)	6(5%)	16(12%)
Grade 3	134	108(81%)	14(10%)	12(9%)
missing	5	3(60%)	0(0%)	2(40%)
**Nodal status**				
Negative	214	173(81%)	13(6%)	28(13%)
Positive	115	96(83%)	8(7%)	11(10%)
missing	1	0(0%)	1(100%)	0(0%)
**LVI**				
Negative	241	194(80%)	17(7%)	30(12%)
Positive	75	64(85%)	5(7%)	6(8%)
missing	14	11(79%)	0(0%)	3(21%)
**Tumour size (group)**				
<=2cm	194	153(79%)	13(7%)	28(14%)
>2-5cm	114	99(87%)	8(7%)	7(6%)
>5cm	21	16(76%)	1(5%)	4(19%)
missing	1	1(100%)	0(0%)	0(0%)
**ER (IHC)**				
Negative	65	55(85%)	5(8%)	5(8%)
Positive	261	212(81%)	17(7%)	32(12%)
missing	4	2(50%)	0(0%)	2(50%)
**PR (IHC)**				
Negative	105	87(83%)	7(7%)	11(10%)
Positive	217	178(82%)	15(7%)	24(11%)
missing	8	4(50%)	0(0%)	4(50%)
**HER2 (IHC)**				
Negative	298	244(82%)	17(6%)	37(12%)
Positive	19	15(79%)	4(21%)	0(0%)
missing	13	10(77%)	1(8%)	2(15%)
**Intrinsic subtype (IHC)**				
Lum NOS	5	4(80%)	0(0%)	1(20%)
Lum A	168	133(79%)	14(8%)	21(13%)
Lum B /Ki67 high	76	66(87%)	0(0%)	10(13%)
Lum B /HER2+	8	6(75%)	2(25%)	0(0%)
HER2+/HR-	11	9(82%)	2(18%)	0(0%)
Basal	29	25(86%)	0(0%)	4(14%)
Additional Basal if by TNP	17	14(82%)	3(18%)	0(0%)
missing	16	12(75%)	1(6%)	3(19%)
**Adjuvant systemic therapy**				
no systemic therapy	78	56(72%)	6(8%)	16(21%)
TAM only	95	81(85%)	5(5%)	9(9%)
chemotherapy only	60	51(85%)	4(7%)	5(8%)
chemotherapy + TAM	96	80(83%)	7(7%)	9(9%)
ovarian ablation or hormonal therapy other than TAM + chemotherapy	1	1(100%)	0(0%)	0(0%)
missing	0	0(0%)	0(0%)	0(0%)

The study cohort included 330 female patients with primary breast cancer originally diagnosed between **January** 1, 1998 and **December** 31, 2002, accessioned in the **Pathology Department at University of British Columbia Hospital** from 1997 to 2002, and who were referred to the **British Columbia Cancer Agency** for treatment. The median follow-up was 13 years.

LVI – lymphovascular invasion; Lum – luminal; HR – hormone receptor; TNP – triple-negative phenotype; TAM – tamoxifen

**Table 2:** Relation between ATG4B and patient characteristics

Patient characteristics	Total	ATG4B cytoplasmic score negative	ATG4B cytoplasmic score any positive	missing	Association/correlation test
**Total**	330	269 (82%)	22 (7%)	39(12%)	
**Grade**					Kendall correlation tau = 0.12 P = 0.0359
Grade 1 or 2	191	158(83%)	8(4%)	25(13%)	
Grade 3	134	108(81%)	14(10%)	12(9%)	
missing	5	3(60%)	0(0%)	2(40%)	
**HER2 (IHC)**					Kendall correlation tau = 0.14 P = 0.0204
Negative	298	244(82%)	17(6%)	37(12%)	
Positive	19	15(79%)	4(21%)	0(0%)	
missing	13	10(77%)	1(8%)	2(15%)	

This table shows the association between tumor grade, HER2 status and ATG4B positive expression

To further investigate ATG4B levels in HER2 positive tumors in vivo, we analyzed BT-474 cell line xenograft tumors from mice treated with different trastuzumab dose regimens: single low (4mg/kg) or high (20mg/kg) dose, or multiple doses (10mg/kg given four times over one week). We compared drug distribution (Figure 6B) and ATG4B levels (Figure 6C) in tumors following these different dosing regimens. Consistent with our observations in vitro, there was a significant reduction in ATG4B levels in trastuzumab-treated tumors (single high dose and multiple-dose groups) compared to controls. An additional HER2 positive cell line xenograft tumor model, JIMT1, was treated with trastuzumab and showed similar effects on ATG4B levels (Figure 6D, 6E). Together, these results show that ATG4B levels in HER2 positive tumors are altered by trastuzumab in a dose-dependent manner in vivo.

## DISCUSSION

Our study is the first to show ATG4B inhibition as a strategy to sensitize HER2 positive breast cancer cells to anti-HER2 therapy (trastuzumab). Moreover, ATG4B inhibition appeared effective in all tested HER2 positive cell lines, independent of the initial sensitivity or resistance to trastuzumab. Although trastuzumab in combination with chemotherapy is currently considered one of the most effective treatments in oncology, many patients with HER2-overexpressing breast cancer develop *de novo* or acquired resistance [37, 38]. Our results suggest that ATG4B inhibition in combination with anti-HER2 therapy can be considered a potentially effective approach for both trastuzumab-sensitive and -resistant breast cancers.

We showed for the first time, using a panel of HER2-negative and HER2-positive cell lines, that there is a positive correlation between HER2 and ATG4B protein expression. We also observed this association in a small number of breast cancer specimens. This observation from our exploratory IHC analysis requires further validation in an independent patient cohort and a larger number of specimens to confirm clinically significant associations with breast cancer subtypes and tumor grade. In this study, we pursued the *in vitro* and *in vivo* evaluation of this novel association and analyzed the context-dependent roles that ATG4B plays in breast cancer cells.

Under baseline conditions, we found that HER2-overexpressing cells had low autophagic activity, but under stress (e.g. starvation, HER2 knockdown), there was an upregulation of autophagy. It was previously shown that HER2 upregulates mTOR[43] – one of the major negative regulators of autophagy [44]. In addition, Negri et al.[45] demonstrated a significant association between *ERBB2/HER2* oncogene amplification and loss of *ATG6/BECN1* (both on 17q21) — a haploinsufficient tumor suppressor that codes for a phylogenetically conserved protein essential for macroautophagy[7, 46]. Tang et al. [47] also showed that low mRNA expression of *BECN1/Beclin 1* was more common in HER2-enriched breast cancers. Together, these findings can explain low baseline autophagy levels in HER2-overexpressing cells.

In contrast to baseline conditions, HER2 inhibition with either HER2-siRNA or trastuzumab, similar to starvation, induced autophagic flux. A number of publications previously showed upregulation of autophagy in response to anti-HER2 therapy [39, 40, 48–50]. Tang et al[48] demonstrated that autophagy induced by lapatinib was mediated by inhibition of the class I PI3K/Akt/mTOR pathway. Alternatively, a direct interaction between Beclin 1 and HER2, described by Han et al. [51] could be involved. Their data suggested that the HER2-Beclin 1 complex is present at the cell surface of HER2-expressing breast cancer cells and this complex is disrupted by lapatinib treatment, which concomitantly induces adaptive autophagy in lapatinib-resistant breast cancer cells. Together, these findings suggest that multiple, including mTOR-independent, mechanisms can play a role in upregulation of autophagy in response to HER2 inhibition.

High ATG4B levels may contribute to both conditions, low autophagic activity in non-stressed cells, and increased autophagic flux in starved and HER2-knockdown cells. It is possible that without stress, in HER2-overexpressing cells, an excessive recycling function of overexpressed ATG4B interferes with proper autophagosome closure and eventually suppresses autophagic flux [24, 52], while under stress, high levels of ATG4B allows autophagy induction and bolsters cell survival. Indeed, HER2-positive ATG4B-deficient cells were unable to upregulate autophagy in response to stress (starvation) and had significantly decreased viability (Figure 3). Strikingly, this viability effect was not observed in the HER2-negative cells, indicating that ATG4B has context-dependent roles in different cancers. A similar decrease in cell viability and proliferation under starvation following ATG4B inhibition was observed in osteosarcoma [16] and colorectal cancer [32] cells; however, in the colorectal cancer model, the growth arrest induced by silencing ATG4B was independent of autophagic flux [32]. Here, the context-dependent role of ATG4B was observed in different breast cancer subtypes and linked to autophagy: HER2-positive, as opposed to HER2-negative, breast cancer cells required ATG4B to upregulate pro-survival autophagy in response to starvation.

In addition, we showed for the first time that other ATG4 family members, especially ATG4C, play a role in autophagy upregulation following HER2 knockdown, possibly compensating for the decrease in ATG4B levels related to HER2 inhibition. ATG4 homologs were previously reported to have selective preferences toward diverse Atg8 family substrates [31], and ATG4C was shown to be required for a proper autophagic response under stressful conditions such as prolonged starvation [53]. Further studies to better understand the activity of different ATG4 family members in the setting of HER2 inhibition are required to find the most effective strategy to target ATG4 family members in combination with targeting HER2.

While development of small molecule inhibitors of ATG4B is still in progress [15, 21], we used a genetic strategy to evaluate the effects of a combination treatment approach in three HER2 positive cell lines. Combination knockdown of HER2 and ATG4B caused a dramatic decrease in cell viability, as well as an inability of cells to upregulate autophagy, confirming that ATG4B is required for pro-survival autophagy in HER2-positive cells. Importantly, we showed that nearly complete, rather than partial, depletion of ATG4B is crucial in order to obtain improved cytotoxicity.

Treatment with trastuzumab (Herceptin) in combination with ATG4B siRNA-mediated knockdown in Herceptin-sensitive and -resistant breast cancer cells significantly reduced cell viability compared to trastuzumab alone in all tested cell lines. Bearing in mind the extracellular actions of trastuzumab, including the immunological control of neoplastic cells [54], it is of great importance to further validate the described effects using appropriate *in vivo* models. As a first step towards future efficacy studies, we validated the effects of trastuzumab on ATG4B levels in two different *in vivo* models and found a significant reduction in ATG4B protein expression following trastuzumab treatment. The significant reduction in cell viability following combination inhibition of ATG4B and HER2 *in vitro*, as well as the positive association between ATG4 protein level and HER2 status *in vivo*, could serve as proof-of-principle for testing clinically relevant inhibitors of HER2 in combination with ATG4B inhibitors in the future.

In conclusion, we showed, for the first time, a functional association between ATG4B and HER2 in breast cancer cells. We explored the possible roles that ATG4B plays in HER2-positive cells and found that ATG4B is required for upregulation of pro-survival autophagy under HER2 inhibition and in nutrient-deprivation conditions. Our results can serve as a proof-of-principle for combination treatment approaches and should be taken into consideration for further *in vivo* validation once suitable ATG4B inhibitors become available.

## MATERIALS AND METHODS

### Reagents

Anti-LC3B (#ab48394, Abcam), anti-β-actin (#ab6276, Abcam), anti-HER2 (#2242L Cell Signaling Technology), anti-ATG4B (#A2981, Sigma), anti-ATG4A (#ARP42722_P050, Aviva Systems Biology), anti-ATG4C (#A9482, Sigma), anti-ATG4D (#ABC22, EMD Millipore), anti-Beclin1 (#NB500-249, Novus Biologicals), anti-ATG5 (#2630, Cell Signaling Technology), anti-ATG7 (#NB110-55474, Novus Biologicals), anti-p62 (#P0067, Sigma), goat anti-mouse IgG–horseradish peroxidase (HRP), and goat anti-rabbit IgG–HRP (Santa Cruz Biotechnology) antibodies were used in immunoblotting and immunohistochemistry. The following drugs were used: Bafilomycin A1 (Sigma-Aldrich), and trastuzumab (Herceptin; Genentech Inc., San Francisco, CA).

### Cell lines and culture conditions

A panel of HER2-negative cell lines included MCF7, MDA-MB-231, SUM159PT, SUM149PT, and Hs 578T. A panel of HER2-overexpressing (positive) cell lines included SKBR3, JIMT1, BT474, MDA-MB-361, and MDA-MB-453. HER2-expressing MCF7 cells (HER2+MCF7) were a gift from Dr. M. Alaoui-Jamali (McGill University, Montreal, Quebec, Canada [55]). Human brain metastatic breast cancer cell lines MDA-MB-231-BR-eGFP and HER2-expressing counterpart HER2+MDA-MB-231-BR-eGFP were developed in the laboratory of Dr Patricia Steeg at the National Cancer Institute (Bethesda, MA)[56].

MDA-MB-231, Hs 578T, BT474, MDA-MB-361, and MDA-MB-453 cells (American Type Culture Collection, ATCC), as well as JIMT1 cells (German Collection of Microorganisms and Cell Culture (Deutsche Sammlung von Mikroorganismen und Zellkulturen GmbH), DSMZ) were maintained in Gibco DMEM (Life Technologies) supplemented with 10% fetal bovine serum (FBS). MCF7 cells (ATCC) and HER2+MCF7 cells were grown in Gibco RPMI-1640 medium with 10% FBS; G418 (1ug/ml) was added to HER2+MCF7 cells weekly. SUM159PT and SUM149PTcells (Asterand) were maintained in Ham's F-12 medium with 10% FBS, HEPES (Sigma-Aldrich), 5 μg/mL insulin (Sigma-Aldrich), and 1 μg/mL hydrocortisone (Sigma-Aldrich). SKBR3 cells (ATCC) were maintained in McCoy's 5A (modified) medium with 10% FBS. SKBR3 cells (ATCC) stably transfected with mRFP-eGFP-LC3B were grown in DMEM supplemented with 10% FBS, 20mM HEPES, and 1X non-essential amino acids (Gibco) with Geneticin (G418). All cells were maintained at 37°C with 5% CO_2_ and 95% humidity.

### Cell viability assays

To evaluate cell viability following ATG4B-siRNA and HER2-siRNA treatment, we used Alamar Blue–based metabolic assay (Invitrogen). Cells were plated at 2×10^4^ cells per well in a 96-well plate and the next day transfected with siRNA as described below. Alamar Blue was added according to the manufacturer's recommendations 48 hours after the second siRNA treatment, and readings taken using an Infinite M1000 (TECAN). Data for cell viability evaluation are presented as percentages relative to the vehicle-only control (mean ± SEM) and graphed using Prism 6.0 (GraphPad Software Inc.).

Cell viability was also determined by the Trypan blue exclusion assay, using a Countess Automated Cell Counter (Life Technologies).

### Crystal violet proliferation assay

Cells were fixed with 4% paraformaldehyde (PFA) and stained with 0.1% crystal violet. Images of the fixed stained cells were captured with a Bio-rad ChemiDoc Imaging system. Retained crystal violet stain was resolubilized in 10% acetic acid. Readings were taken at A_590_ using a plate reader (VersaMAX Microplate Reader).

### siRNA transfection

Cells were plated at 4 × 10^5^ cells per well in a 6-well plate in serum-free medium and transfected with 75 pmol ATG4B or HER2 siRNAs or a scramble medium GC siRNA negative control (Invitrogen) using Lipofectamine RNAiMAX^TM^ (Invitrogen) as per manufacturer's recommendations. Serum-free medium was replaced with fresh media containing 10% FBS the next day. Forty-eight hours after the initial siRNA transfection, cells were transfected again with respective siRNAs following the same protocol. Seventy-two hours after the second transfection the cells were harvested. For the experiments involving starvation, forty-eight hours after the second transfection the medium in respective wells was replaced with Earl's Balanced Salt Solution (EBSS). After twenty-four hours under either starvation or fed conditions (i.e. seventy-two hours after the second transfection) the cells were either harvested for western blot, or fixed with formalin for crystal violet proliferation assay.

### SKBR3-mRFP-eGFP-LC3B (SKBR3-tfLC3) assay

SKBR3 cells (ATCC) stably transfected with mRFP-eGFP-LC3B [57] were plated at a density 1,600 cells per well in 200uL of DMEM with G418 in 8-well chamber slides. Twenty-four and seventy-two hours later cells were transfected with ATG4B-siRNA, ATG4A-siRNA, ATG4C-siRNA, ATG4D-siRNA, HER2-siRNA, or medium GC-siRNA constructs. Media was replenished every day after initial plating and before siRNA treatment. Complete starvation was performed by incubation in PBS for two hours at 37°C. At the end of the experiment the cells were fixed in 4% PFA for 20 minutes and then rinsed with PBS. Cells were then mounted using SlowFade Gold Antifade with DAPI (Invitrogen). All cells were imaged on a Leica TCS SP8 inverted confocal microscope with a Leica HC PL APO 63x/1.40 oil objective and LAS AF software (Leica). The pinhole and laser brightness settings were kept constant by applying the same properties between comparable experiments. For each treatment, the number of red and yellow puncta per cell were determined for at least 100 cells. Puncta counting was performed using the OpenCV Python package; a contours discovery algorithm[58] was applied to images pre-processed by filtering colors and applying a Gaussian filter and adaptive threshold.

### Western blot analysis

Western blot analysis was performed as described earlier [26, 41]. Briefly, protein lysates were prepared using the RIPA Lysis Buffer Kit (Santa Cruz Biotechnology), according to the manufacturer's protocol. Electrophoresis and transfer of proteins were performed using standard methods and protein–antibody complexes were detected by chemiluminescence assays. Quantitation of the signal was performed using Bio-Rad Image Lab analysis software. The autophagic flux assay was performed using saturating concentrations (20-40 nM) of lysosomal inhibitor Bafilomycin A1.

### Caspase-Glo 3/7 assay (Promega)

HER2+MCF7 cells (8 × 10^3^) were plated in each well of 96-well optical plates (BD Biosciences) and transfected sequentially on day 2 and 4 with scramble or ATG-siRNAs (5 pmol). Cycloheximide served as a positive control. Forty-eight hours after the second transfection, cells in respective wells were starved with EBSS for twenty-four hours, followed by caspase activity assessment with the Caspase-Glo 3/7 Kit. Luminescence was measured with a Synergy H4 Hybrid (BioTek).

### Study population

The study cohort included 330 female patients with primary breast cancer originally diagnosed between January 1, 1998 and **December** 31, 2002, accessioned in the Pathology **Department** at University of British Columbia (UBC) **Hospital** from 1997 to 2002, and who were referred to the BC Cancer Agency for treatment. Cases with micro-invasive primary tumors whose tissue blocks did not contain sufficient material for tissue microarray construction were excluded. Patients with a previously diagnosed breast cancer, or synchronous breast cancer (additional diagnosis of breast cancer within 6 months of diagnosis of the primary) were excluded. The median follow-up was 13 years. Ethical approval for this study was obtained from the Clinical Research Ethics Board of the British Columbia Cancer Agency, and Simon Fraser University.

Patient characteristics are presented in Table 1.

### Immunohistochemistry, scoring, and analysis of markers

Slides from formalin fixed paraffin embedded archival blocks from the 330 patients were stained with hematoxylin and eosin (H&E). These slides were reviewed by a pathologist to identify areas of invasive breast carcinoma. Two 0.6 mm cores from each block were extracted and embedded into three tissue microarray blocks. Slices from this tissue microarray were stained previously with H&E, ER, PR, HER2, Ki67, CK5/6 and EGFR using procedures as described [59, 60]. Intrinsic subtypes based on immunohistochemical marker panel were assigned using the definitions previously described [59, 60]. ATG4B immunohistochemistry was performed using the Ventana Discovery Ultra automated instrument (Ventana Medical Systems Inc, Tuscon, USA) according to manufacturer's recommendations.

All clinical and pathological data for each tumor sample was blinded to the pathologist during scoring. Each sample was assessed for the presence (i.e. positivity) of diffuse ATG4B under X50 magnification.

### Statistical analysis

Statistical analysis was performed using R-3.2.1. Clinicopathological parameter and biomarker associations were assessed using contingency tables. The significance of associations was determined using Fisher's exact test for parameters that are nominal such as intrinsic subtype. The significance of correlations was determined using Kendall's tau test for parameters that are ordinal such as tumor grade.

The cut point (negative vs. any positive) for ATG4B was chosen to maximize statistical power. Distribution of the scores was assessed using frequency tables and histograms. The cut point that resulted in the minimal difference between the number of positive and negative cases was chosen.

All immunohistochemistry tissue microarray images and scores used in this study are publicly available at the companion site http://www.gpecimage.ubc.ca (username: atg4b; password: abc123).

### *ATG4* gene expression analysis

Patient status for *ERBB2* DNA alteration (copy number alteration or mutation), mRNA overexpression (Z-score > 2.0), and protein overexpression (Z-score > 2.0) were obtained from cbioportal.org [61, 62] for The Cancer Genome Atlas (TCGA) invasive breast cancer data set (BRCA) [63]. Quantile normalized Level 3 mRNA levels (illuminahiseq_rnaseqv2-RSEM_genes_normalized) and Level 3 protein levels (mda_rppa_core-protein_normalization) were downloaded from firebrowse.org for these same TCGA BRCA patients. Gene abundance for genes of interest were stratified by patient DNA alteration status (amplified or mutant or wild type; n=959) or PAM50 subtype (n=579) for patients where status was available in cbioportal.org. Gene abundance was also stratified by ERBB2/HER2 status, where we defined HER2 positive patients (HER2+) as patients for which ERBB2 protein levels had been assessed by RPPA platform (n=410), who were found to meet the following parameters: 1) ERBB2 protein overexpression (Z-score > 2.0), 2) *ERBB2* mRNA overexpression (Z-score > 2.0), or 3) *ERBB2* amplification. Many patients showed a combination of these conditions: ERBB2 amplification/mRNA overexpression (OE)/protein OE (n=25), ERBB2 amplification/mRNA OE (n=27), amplification only (n=7), mRNA OE only (n=12), mRNA OE/protein OE (n=1). To examine ATG4B and ERBB2 co-expression, we plotted ATG4B mRNA levels (Level 3 normalized RSEM Z-scores) versus ERBB2 protein levels (Level 3 normalized RPPA Z-scores) and calculated the Spearman correlation for HER+ patients and HER2- patients.

### In vivo studies

Mice bearing BT-474 and JIMT1 xenograft tumors received intraperitoneal injections of unlabeled trastuzumab at 4, 10 or 20 mg/kg and tumors collected at 4-24h after the last administration. An intravenous injection of 0.6mg/ml DioC_7_(3), a fluorescent carbocyanine dye labeling patent vessels, was administered 5 minutes prior to tissue collection and cryopreservation. Cryosections (10 μm) were imaged directly for native DioC_7_(3) fluorescence and then stained for bound trastuzumab using fluorescently tagged antihuman secondary antibodies and re-imaged, as previously described [64]. ATG4B and actin protein levels were assessed by western blot analysis; protein extraction procedure has been previously described in [41].

## SUPPLEMENTARY MATERIAL FIGURES


