# Supplementary material for: Identification of breast cancer cell subtypes sensitive to ATG4B inhibition
Source: Oncotarget. 2016 Aug 19;7(41):66970–88. doi: 10.18632/oncotarget.11408 (PMC5341851; doi:10.18632/oncotarget.11408)
Supplement: Supplementary file 1 [file oncotarget-07-66970-s001.pdf]

Identification of breast cancer cell subtypes sensitive to ATG4B inhibition

SUPPLEMENTARY FIGURES

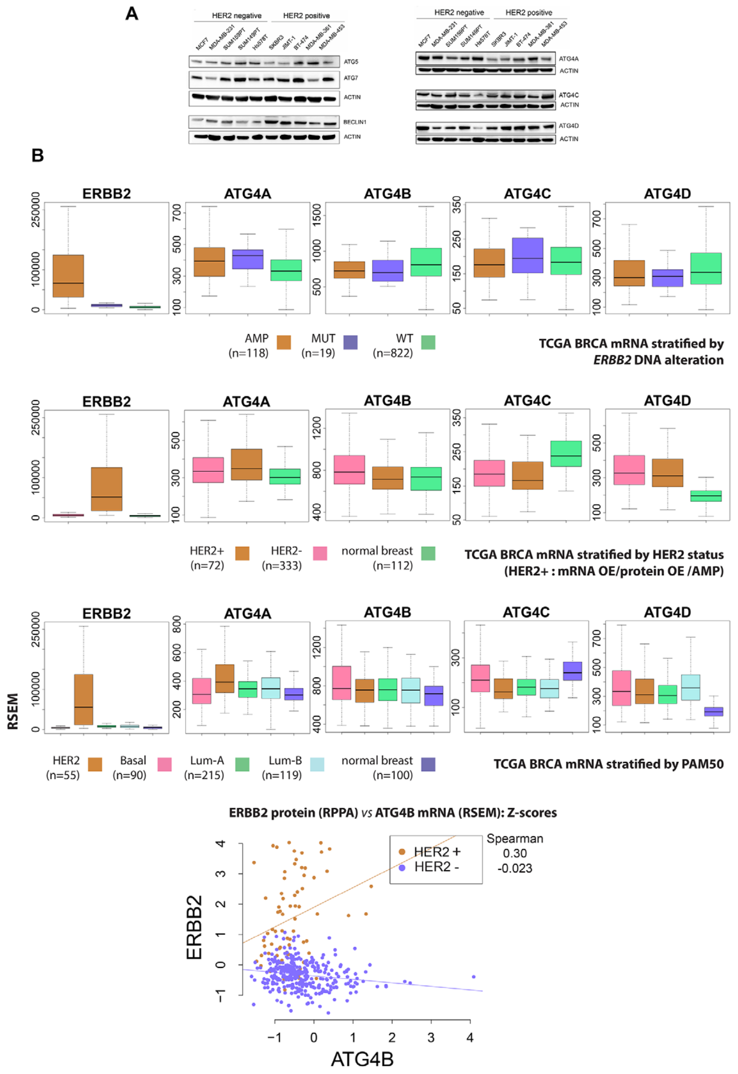

Supplementary Figure S1: ATG expression analysis. A. The expression levels of autophay-related proteins are variable in HER2-positive and HER2-negative cell lines. On the left, representative western blot analysis shows ATG5, ATG7, and Beclin1 basal expression in a panel of HER2-positive (n=5) and HER2-negative (n=5) breast cancer cell lines. On the right, representative (n=2) western blot analysis shows ATG4A, ATG4C, and ATG4D basal expression in a panel of HER2-positive (n=5) and HER2-negative (n=5) breast cancer cell lines. **B.** *ATG4* gene levels stratified by *ERBB2/HER2* status in breast cancer patients sequenced by The Cancer Genome Atlas (TCGA) consortium. RNA-seq derived mRNA levels for ATG4 paralogs (Level 3 normalized RSEM) in patients with invasive breast carcinoma (BRCA) sequenced by TCGA consortium are stratified by patient status for the following: *ERBB2* DNA alteration, *HER2* status, or PAM50 subtype. DNA alterations included amplifications (AMP) or mutations (MUT). HER2-positive status (HER2+; n=72) included patients assessed for protein levels of ERBB2 by RPPA platform (n=410), who showed *ERBB2* mRNA overexpression (mRNA OE) or ERBB2 protein overexpression (protein OE) with Z-scores > 2.0, or *ERBB2* amplification, or combinations of these categories (n=72). ERBB2 protein levels (Level 3 normalized RPPA Z-scores) are plotted versus ATG4B mRNA levels (Level 3 normalized RSEM Z-scores), with HER2 positive (HER2+) patients showing a mild correlation (Spearman r=0.30) versus HER2 negative (HER2-) patients with no correlation (Spearman r=-0.023).

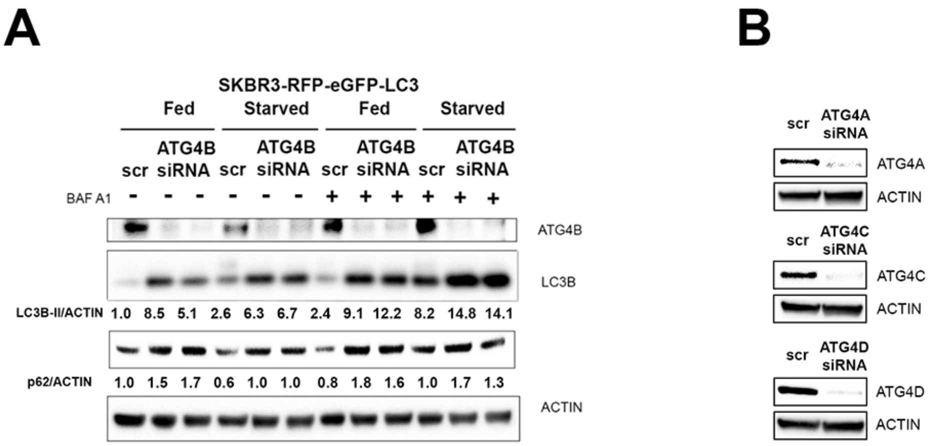

Supplementary Figure S2: Protein changes following knockdown of ATG4 family members. A. ATG4B knockdown results in inhibition of autophagy. Representative western blot flux assay using saturating concentrations of Bafilomycin A1 (Baf A1) show LC3B-II and p62 levels in SKBR3-tfLC3B cells following ATG4B knockdown under fed and starved conditions. **B.** Representative western blot shows decreased ATG4A, ATG4C, and ATG4D protein levels 72 hours after siRNA treatment.
